# Shifting Trends of Antimicrobial Resistance Patterns Among Uropathogenic Bacteria Before and During the COVID-19 Pandemic

**DOI:** 10.7759/cureus.73267

**Published:** 2024-11-08

**Authors:** Hadi B AlHemsi, Ibraheem Altamimi, Abdulaziz Altamimi, Hadeel B Alhemsi, Ibrahim M Alabdulkarim, Alia Zawawi, Abdulrahman Almugren, Abdullah Alhumimidi, Maee Barakeh, Mohamed Y Alquhidan, Fatimah Alshahrani, Mohamed-Hani Temsah, Abdullah Altamimi

**Affiliations:** 1 College of Medicine, King Saud University, Riyadh, SAU; 2 College of Medicine, King Saud Bin Abdulaziz University for Health and Sciences, Riyadh, SAU; 3 College of Medicine, Alfaisal University, King Faisal Specialist Hospital, Riyadh, SAU; 4 Medicine, Division of Infectious Diseases, Department of Internal Medicine, King Saud University Medical City, King Saud University, Riyadh, SAU; 5 Medicine, College of Medicine, King Saud University, Riyadh, SAU; 6 Pediatric Emergency and Medical Toxicology, King Saud University/ King Fahad Medical City, Riyadh, SAU; 7 Evidence-Based Health Care and Knowledge Translation Research Chair, King Saud University, Riyadh, SAU; 8 Pediatric Emergency, King Fahad Medical City, Riyadh, SAU

**Keywords:** antimicrobial resistance (amr), antimicrobial stewardship, covid-19, multidrug resistance (mdr), uropathogenic bacteria

## Abstract

Urinary tract infections (UTIs) represent a significant global health concern, primarily caused by uropathogenic bacteria and their rising trend of antimicrobial resistance (AMR). This study investigates the prevalence and pattern of AMR among uropathogenic bacteria during the COVID-19 pandemic, highlighting its impact on antimicrobial usage and resistance trends. This retrospective cross-sectional study, conducted at King Fahad Medical City, Riyadh, Saudi Arabia, from January 1, 2018, to December 31, 2022, analyzed 10,031 patients with positive urine cultures for resistance patterns pre-COVID-19 and during COVID-19. Bacterial identification and antimicrobial susceptibility testing were performed using the BD Phoenix system according to Clinical Laboratory Standard Institute guidelines. This study recorded an overall decrease in AMR during the pandemic among the most prevalent uropathogens (*Escherichia coli*, *Klebsiella pneumoniae*, and *Pseudomonas aeruginosa*); however, notable increases in AMR were observed for specific antibiotics like cefoxitin, imipenem, and colistin. resistance. The multidrug resistance (MDR) among *P. aeruginosa* strains significantly decreased from 22.9% pre-pandemic to 9.2% during the pandemic. The decline in AMR patterns during the COVID-19 pandemic likely resulted from altered antibiotic usage and healthcare practices, emphasizing the importance of ongoing monitoring and targeted antimicrobial management in response to changing AMR dynamics during global health emergencies.

## Introduction

Every year, outpatient and long-term care facilities globally detect between 150 and 250 million cases of urinary infections (UTIs), making it a major health concern. These infections affect people from all age groups, which impacts society through increased costs of care and work absenteeism. Significant consequences of UTIs include recurrent episodes, pyelonephritis and cystitis in young children, premature birth, and the emergence of high-level antibiotic resistance due to frequent antimicrobial usage [1]. 

Uropathogenic bacteria are the primary cause of UTIs, though other organisms like fungi, viruses, and parasites can also contribute [2]. It is important for healthcare providers to accurately detect uropathogens and their susceptibility to antibiotics. By tailoring their prescription effectively, the risk of antibiotic misuse and its associated repercussions would be minimized. Ideally, the clinician would resort to a holistic approach, which includes taking a full clinical history, performing a physical examination, and diagnosing UTIs through the conventional culture-based method, which remains the gold standard [3]. However, this approach entails an average delay of 2-3 days, which is inadequate for patients facing complicated UTIs, especially those at risk of developing life-threatening urosepsis. In critical scenarios, physicians initiate empiric antibiotic therapy. If the chosen antibiotic regimen is insufficient, they make adjustments based on their knowledge of local pathogen prevalence and antibiogram profiles [4]. This ensures an optimal outcome for the patient while combating the growing threat of antibiotic resistance in healthcare settings.

UTIs are commonly caused by Enterobacteriaceae like *Escherichia coli (E. coli)* and* Klebsiella species (sp.)*, along with other Gram-negative bacteria. These infections represent a leading cause of febrile illnesses in children [5]. The emergence of extended-spectrum β-lactamase (ESBL)-producing Enterobacteriaceae in UTIs has been correlated with the administration of broad-spectrum antibiotics, exacerbating the challenge of antimicrobial resistance (AMR) [5, 6]. A recent study identified the common bacterial uropathogens and determined their prevalence of AMR, highlighting* E. coli *(62.94%) and *Klebsiella pneumoniae (K. pneumoniae) *(12.35%) as the predominant resistant bacterial strains [7].

The COVID-19 pandemic has placed unprecedented strain on global healthcare systems and has resulted in a significant increase in antibiotic usage, further complicating the challenge of AMR [8]. Recent studies have provided valuable insights into the dynamics of AMR trends during the pandemic. A study at a large tertiary care center revealed no significant changes in antimicrobial susceptibility during the COVID-19 pandemic.* E. coli *was mostly sensitive to meropenem and ertapenem in 99.2% of cases, while *Klebsiella sp. *was sensitive to amikacin in 97.1% of cases [9]. However, other findings revealed contrasting trends during the pandemic, noticing a decline in extended-spectrum β-lactamase (ESBL) and carbapenem resistance among uropathogens. ESBL production decreased in* E. coli *by 1.9% and in* K. pneumoniae* by 6.0%. Moreover,* E. coli *exhibited a 1.2% reduction in carbapenem resistance, *K. pneumoniae* decreased by 10.7%, and* P. aeruginosa* decreased by 7.9% [7]. Similar findings were reported by another study; however, it notably showed higher resistance to certain antibiotics, specifically trimethoprim treatment used as empirical therapy, in hospitals (42.6%) and aged care facilities (38.8%) compared to those isolated from the community (30%) [10]. In 2023, two published studies reported a significant increase in uropathogenic bacterial resistance profiles before and after the COVID-19 pandemic, particularly regarding *E. coli’s* resistance to amoxicillin and levofloxacin and *Klebsiella sp*. to amoxicillin and ceftriaxone. Remarkably, *Klebsiella sp. *showed a decreased resistance to amikacin, carbapenems, and trimethoprim-sulfamethoxazole [11, 12]. Moreover, a comparative study in Iran between 2020 and 2022 highlighted a significant resistance increase for *E. coli *to ampicillin, carbapenems, and ceftazidime [13]. There is considerable heterogeneity in the multidrug resistance (MDR) and AMR of uropathogenic bacteria, especially after increased antibiotic use and systemic disturbances during the pandemic.

There are gaps in our knowledge about the frequency and trends of AMR among uropathogenic bacteria both before and after the pandemic, as well as the effects of infection control strategies and modifications to antibiotic prescribing practices. This study aims to explore the prevalence and patterns of AMR among uropathogenic bacteria over the course of the COVID-19 pandemic. This forward-looking direction is crucial for gaining a deeper and more precise understanding of AMR trends, especially in the context of unprecedented health challenges such as the COVID-19 pandemic.

## Materials and methods

Study design, setting, and duration

The study was conducted at King Fahad Medical City (KFMC), a renowned tertiary hospital in Riyadh, Saudi Arabia. The retrospective study received approval from the Institutional Review Board (IRB) of KFMC (IRB00010471). KFMC is a prominent referral center for patients from various local health centers, private hospitals, and clinics. Covering the period from January 1, 2018, to December 31, 2022, this retrospective observational study aimed to comprehensively analyze all positive urine samples submitted for culture and sensitivity testing during this timeframe. To assess the impact of the COVID-19 pandemic accurately, a specific timeline was established based on the emergence of the first identified case [14]. Urine cultures identified before March 3, 2020, were classified as 'pre-COVID-19,' representing a period unaffected by the pandemic’s influence. This classification was crucial for evaluating pandemic response measures and strategies. Urine cultures identified on March 3, 2020, or after, were categorized as 'during COVID-19,' signifying the onset and continuation of the pandemic [7,15]. This temporal classification was essential for an accurate evaluation of the pandemic's progression and the effectiveness of implemented strategies.

Inclusion and exclusion criteria

Participants with suspected UTIs were selected based on the following inclusion criteria: fever and urinary symptoms such as dysuria, hesitancy, frequency, urgency, low-volume voids, or discomfort in the lower abdomen. For infants and children, a UTI was identified if a properly collected urine sample showed a single pathogen exceeding a threshold of 10 × 105 CFU/mL, along with urinary symptoms [9-17]. The study focused on patients whose urine cultures tested positive for* E. coli, K. pneumoniae, and P. aeruginosa.* Only urine samples with a significant microbial load above 105 CFU/mL were included to ensure a thorough characterization of infections [18]. Exclusion criteria were employed to maintain the reliability and validity of the study. These criteria excluded urine samples with multiple types of bacteria, samples collected more than three days after hospital admission, and samples from patients with chronic renal failure, severe birth defects, or those collected using pediatric bags [9,19]. This research adhered to the European guidelines for sample registration, documentation, and transportation to the microbiological laboratory. Proper protocols for sample handling, storage, and refrigeration were implemented to preserve sample quality [19,20]. These criteria aimed to ensure the study's reliability, accuracy, scientific rigor, and validity.

Approach to culturing and examining urine specimens: compliance with WHO standards using semi-quantitative methods

The methodology for cultivating and analyzing urine specimens adhered to the World Health Organization's (WHO) suggested protocols, employing a semi-quantitative approach [21]. Each urine specimen, precisely 1 mL, was evenly distributed on cystine-lactose-electrolyte-deficient (CLED) as well as blood agar plates, sourced from Hardy Diagnostics in Santa Maria, CA, USA [22]. Following inoculation, the plates were aerobically incubated at a temperature of 37°C for a period of 24 hours, using a standardized wire loop for calibration. The identification and characterization of the microbial isolates were achieved through an integrated approach, combining biochemical tests with an examination of the growth characteristics. Analytical systems like the BD Phoenix (supplied by BD-Canada, Mississauga, ON, Canada) and the API ID system (provided by bioMerieux UK Ltd., Basingstoke, UK) were employed in these analyses [23].

Application of the BD Phoenix system in bacterial isolate identification and antimicrobial susceptibility testing

The BD Phoenix system's NMIC/ID-4 panel was utilized to analyze 10031 bacterial isolates. A stock solution with a McFarland standard of 0.5 was prepared using 4.5 mL of Phoenix ID broth and a Sensi Titre™ nephelometer from Thermo Fisher Scientific, Waltham, MA, USA [24]. This bacterial identification solution was allocated to the appropriate ID area on the Phoenix panel. Each chemical reactivity well on the panel received 50 µL of the bacterial solution. The sealed panel, along with its code, was then inserted into the Phoenix machine for processing. The identification results obtained from the Phoenix system were compared with those from the conventional API system to assess identification accuracy at both the genus and species levels [25].

Adherence to CLSI guidelines and identification of multidrug-resistant isolates

In line with the Clinical Laboratory Standard Institute (CLSI) version 6.0 guidelines, all identified pseudomonas strains were tested for their antimicrobial susceptibility using the agar disc diffusion method, as provided by Hardy Diagnostics in Santa Maria, CA, USA [26]. The antibiotics tested in this study included: ampicillin (10 μg), amoxicillin-clavulanate (20/10 μg), cephalothin (30 μg), cefuroxime (30 μg), ceftazidime (30 μg), cefoxitin (30 μg), cefepime (30 μg), cefotaxime (30 μg), ceftriaxone (30 μg), ciprofloxacin (5 μg), gentamicin (10 μg), amikacin (30 μg), trimethoprim-sulfamethoxazole (1.25/23.75 μg), piperacillin-tazobactam (36 μg), imipenem (10 μg), meropenem (10 μg), ertapenem (10 μg), levofloxacin (5 μg), tigecycline (15 μg), nitrofurantoin (300 μg), and colistin (10 μg).

The analysis of the test results adhered to the diameter or breakpoint suggestions provided by the CLSI, which helped to classify the bacterial isolates into susceptible (S), intermediate (I), or resistant (R) categories. Isolates that exhibited resistance to antibiotics across three or more classes were identified as multidrug-resistant. Through the application of the Kirby Bauer disc diffusion technique, this investigation aimed to determine the resistance patterns of isolates against a comprehensive selection of antibiotics, following the guidelines of the CLSI, and to pinpoint isolates with multidrug resistance [27].

Statistical analysis

Data was extracted onto dedicated Microsoft Excel sheets (Redmond, USA) and exported to IBM Corp. Released 2020. IBM SPSS Statistics for Windows, Version 27.0. Armonk, NY: IBM Corp. for analysis. Categorical outcomes were presented as frequencies and percentages. The singular continuous outcome (age) was presented as mean values with standard deviation. Normal distribution was assessed visually through histogram plots and statistically using the Kolmogorov-Smirnov test for normality. The association between specific antibiotic resistance and time (pre-COVID-19 vs. during COVID-19) was checked using the chi-square test. A logistic regression model was used to test for association of MDR among *P. aeruginosa* strains and categorical variables, and a linear regression model was used to test for association in the case of a continuous variable (age). This study utilized multivariate-adjusted odds ratios (OR) with 95% confidence intervals to evaluate the relationship between dependent variables and participant characteristics. Bar and line graphs constituted the visual representation of data. A p-value of <0.05 was regarded as being statistically significant for all analyses.

## Results

Data for 19,363 patients was obtained, out of which a total of 10,031 patients had their urine samples collected. Out of these urine samples, 6,756 (67.4%) revealed *E. coli *growth, 2,218 (22.1%) revealed *K. pneumoniae* growth, and 1,057 (10.5%) revealed *P. aeruginosa* growth. Regarding baseline demographic characteristics, the minimum age of the participants was 1 day old, and the maximum age was 106 years. The mean age of the overall study population was 47.3 ± 24.7 years. A total of 2,074 patients (20.7%) were under the age of 18. Most samples were derived from inpatients (approximately 96%). Females made up the majority of the study population at 68.9% (n=6,907), while the remaining 31.1% were males (n=3,124). A detailed breakdown of the gender distribution per year for each bacterium has been given in Table 1.

**Table 1:** Baseline characteristics of participants.

Bacteria	Year	Males (n, %)	Females (n, %)
Escherichia coli	2018	9 (40.9)	13 (59.1)
	2019	462 (24.4)	1434 (75.6)
	2020	381 (24.9)	1149 (75.1)
	2021	285 (24.6)	872 (75.4)
	2022	601 (27.9)	1550 (72.1)
	Total	1738 (25.7)	5018 (74.3)
Klebsiella pneumoniae	2018	44 (50.6)	43 (49.4)
	2019	272 (33.9)	531 (66.1)
	2020	195 (36.2)	344 (63.8)
	2021	9 (47.4)	10 (52.6)
	2022	261 (33.9)	509 (66.1)
	Total	781 (35.2)	1437 (64.8)
Pseudomonas aeruginosa	2018	24 (54.5)	20 (45.5)
	2019	193 (60.9)	124 (39.1)
	2020	143 (59.6)	97 (40.4)
	2021	100 (52.6)	90 (47.4)
	2022	145 (54.7)	120 (45.3)
	Total	605 (57.3)	451 (42.7)

**Figure 1:**
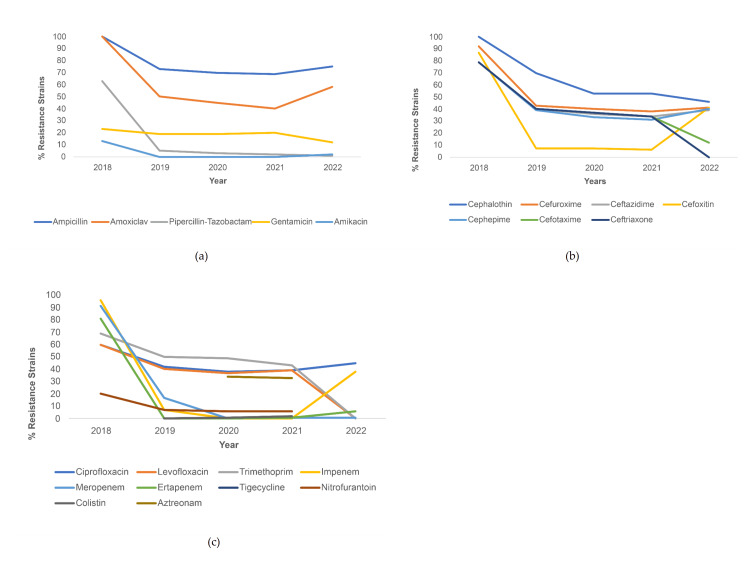
Resistance trends among E. coli strains for (a) penicillin and aminoglycoside antibiotics, (b) cephalosporin antibiotics, and (c) other antibiotics.

In terms of resistance to penicillin antibiotics for *E. coli*, resistance was highest for ampicillin and lowest for piperacillin-tazobactam throughout 2018-2022, and a decreasing trend was noted for all three penicillin antibiotics. Resistance to ampicillin started at 100% in 2018 and ended up being 77% in 2022. Resistance to Amoxiclav started at 100% in 2018, reached its lowest value in 2021 (39%), and rose again in 2022 (47%). A trend of uniformly decreasing resistance was noted for the two aminoglycosides (Figure 1a). For cephalosporins, all of the tested antibiotics demonstrated a decreasing resistance trend, with the exception of ceftazidime, cefuroxime, and cefoxitin. These three cephalosporins saw an increase in resistance from 2021 to 2022, but the increase in resistance was sharpest for cefoxitin (from 10% to approximately 50%). Among these cephalosporins, cephalothin was the antibiotic to which most strains were resistant across each year (Figure 1b). Figure 1c illustrates the trends of resistance seen to other antibiotics. Among these antibiotics, the majority demonstrated a decreasing resistance trend, except for imipenem, resistance to which rose sharply from 2021 (less than 5%) to 2022 (approximately 40%), and both quinolones, which displayed almost steady resistance throughout the study time period (fluctuating between 60% and 45%).

The percentage of *E. coli *strains resistant to individual antibiotics and their association with the timeline (before COVID-19 vs. during COVID-19) have been illustrated in Table 2. Percentage of *E. coli *strains resistant to ampicillin, amoxiclav, piperacillin-tazobactam, cephalothin, cefuroxime, cefotaxime, ceftriaxone, levofloxacin, gentamicin, trimethoprim-sulfamethoxazole, meropenem, and tigecycline decreased during COVID-19, with statistical significance. The greatest decrease in % resistance was seen for trimethoprim-sulfamethoxazole (a decrease of 24.1%). Meanwhile, the only resistance to cefoxitin (7.9% to 23.5%) and imipenem (6.3% to 18.1%) increased during COVID-19 with statistical significance (p<.001).

**Table 2:** Resistance patterns for E. coli before and during COVID-19. * Statistically significant

Antibiotics	Before COVID-19		During COVID-19		p-value
	R (%)	S (%)	R (%)	S (%)	
Ampicillin	72.6	27.4	68.5	31.5	.001*
Amoxiclav	50.5	49.5	40.9	59.1	< .001*
Piperacillin-Tazobactam	5.6	94.4	2.7	97.3	< .001*
Cephalothin	68.3	31.7	48.9	51.1	< .001*
Cefuroxime	43.3	56.7	39.6	60.4	.003*
Ceftazidime	38.5	61.5	36.9	63.1	.187
Cefoxitin	7.9	92.1	23.5	76.5	< .001*
Cefepime	38.0	62.0	36.6	63.4	.244
Cefotaxime	39.8	60.2	24.3	75.7	< .001*
Ceftriaxone	39.8	60.2	18.3	81.7	< .001*
Ciprofloxacin	40.3	59.7	41.5	58.5	.346
Levofloxacin	38.5	61.5	28.7	71.3	< .001*
Gentamicin	14.2	85.8	10.7	89.3	< .001*
Amikacin	0.6	99.4	1.0	99.0	.093
Trimethoprim-Sulfamethoxazole	48.8	51.2	24.7	75.3	< .001*
Imipenem	6.3	93.7	18.1	81.9	< .001*
Meropenem	2.1	97.9	0.8	99.2	< .001*
Ertapenem	1.9	98.1	2.1	97.9	.553
Tigecycline	5.9	94.1	0.4	99.6	.002*
Nitrofurantoin	5.6	94.4	4.5	95.5	.107
Colistin	0	100	0.6	99.4	.138
Aztreonam	38.2	61.8	33.6	66.4	.093

**Figure 2:**
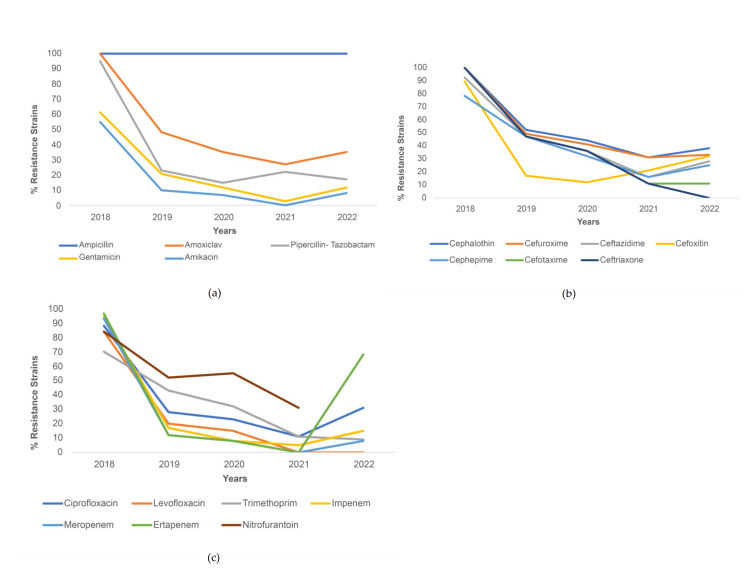
Resistance trends among K. pneumoniae strains for (a) penicillin and aminoglycoside antibiotics, (b) cephalosporin antibiotics, and (c) other antibiotics.

For *K. pneumoniae*, all strains were resistant to ampicillin from 2018 to 2022 (100%). The other penicillin antibiotics and the two tested aminoglycosides saw a decreasing trend from 2018 to 2021 with a slight increase in % resistance from 2021 to 2022, except for piperacillin-tazobactam, which increased from 2020 (18%) to 2021 (25%) but then saw a sharp decline in 2022 (approximately 10%) (Figure 2a). For cephalosporins, all the antibiotics except cefoxitin demonstrated decreased resistance from 2018 to 2022, with the most dramatic decrease seen in ceftriaxone (from 100% in 2018 to 0% in 2022). Resistance to cefoxitin decreased sharply from 2018 (88%) to 2019 (17%), but then saw an increase between 2020 (15%) and 2022 (approximately 30%) (Figure 2b). For the other antibiotics, resistance to both quinolones decreased throughout the study period, but resistance to ciprofloxacin increased sharply from 2021 (16%) to 2022 (30%). This trend of an initial decrease in resistance from 2018 to 2021 but increasing resistance from 2021 to 2022 was also seen among the three carbapenems, but the most dramatic increase was seen for ertapenem (from 0% to approximately 60%) (Figure 2c).

Percentage of *K. pneumoniae* strains resistant to amoxiclav, piperacillin-tazobactam, cephalothin, cefuroxime, ceftazidime, cefepime, cefotaxime, ceftriaxone, ciprofloxacin, levofloxacin, gentamicin, trimethoprim-sulfamethoxazole, imipenem, meropenem, and tigecycline saw a decrease during COVID-19, with statistical significance. The greatest decrease in resistance was seen for ceftriaxone (31.0% decrease). Meanwhile, the percentage of strains resistant to ertapenem and aztreonam increased during COVID-19 (p<.001) (Table 3).

**Table 3:** Resistance patterns for K. pneumoniae before and during COVID-19. * Statistically significant

Antibiotics	Before COVID-19		During COVID-19		p-value
	R (%)	S (%)	R (%)	S (%)	
Ampicillin	100	0	100	0	-
Amoxiclav	50.7	49.3	35.1	64.9	< .001*
Piperacillin-Tazobactam	28.6	71.4	11.0	89.0	< .001*
Cephalothin	54.4	45.6	40.1	59.9	< .001*
Cefuroxime	51.3	48.7	36.2	63.8	< .001*
Ceftazidime	46.6	53.4	31.8	68.2	< .001*
Cefoxitin	22.4	77.6	25.3	74.7	.103
Cefepime	45.2	54.8	28.2	71.8	< .001*
Cefotaxime	48.3	51.7	20.3	79.7	< .001*
Ceftriaxone	48.0	52.0	17.3	82.7	< .001*
Ciprofloxacin	31.8	68.2	27.2	72.8	.017*
Levofloxacin	24.6	75.4	15.4	84.6	< .001*
Gentamicin	23.2	76.8	14.1	85.9	< .001*
Amikacin	12.2	87.8	7.6	92.4	< .001*
Trimethoprim-Sulfamethoxazole	43.2	56.8	16.4	83.6	< .001*
Imipenem	22.1	77.9	12.6	87.4	< .001*
Meropenem	18.0	82.0	8.4	91.6	< .001*
Ertapenem	18.5	81.5	37.4	62.6	< .001*
Tigecycline	33.9	66.1	15.5	84.5	.003*
Nitrofurantoin	54.1	45.9	53.4	46.6	.801
Aztreonam	25.0	75.0	33.8	66.2	< .001*

**Figure 3:**
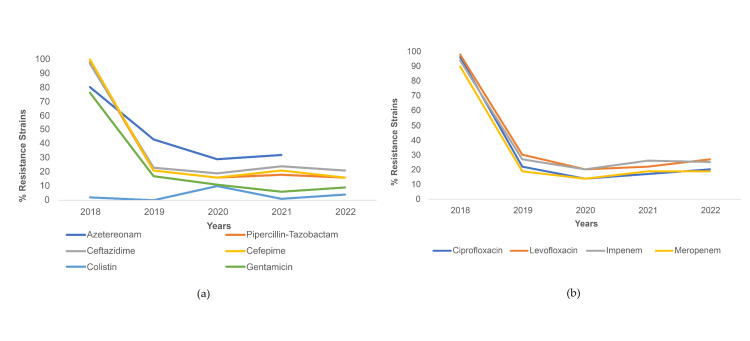
Resistance trends among P. aeruginosa strains for (a) cephalosporins and other antibiotics and (b) fluoroquinolones and carbapenem antibiotics.

Trends of resistance in* P. aeruginosa* throughout the study time period were noted to be similar for piperacillin-tazobactam, cefepime, and ceftazidime (Figure 3a), and ciprofloxacin, levofloxacin, imipenem, and meropenem (Figure 3b). These antibiotics saw a sharp decline in resistance from 2018 to 2019 (declining from resistance > 90% to < 40%), with resistance levels showing a plateau in the subsequent years. Resistance to aztreonam decreased from 45% in 2018 to 10% in 2019 and further decreased to 3% in 2021. A similar trend was seen for gentamicin, with a decrease from 76% resistance in 2018 to 18% in 2019, and 11% in 2021 (Figure 3a). Meanwhile, contrary to other trends, resistance to colistin rose from 0% in 2019 to 14% in 2020 (Figure 3a).

Percentage of *P. aeruginosa* strains resistant to piperacillin-tazobactam, ceftazidime, cefepime, ciprofloxacin, levofloxacin, gentamicin, amikacin, imipenem, meropenem, and aztreonam decreased during COVID-19 with statistical significance. The greatest decrease in % resistance was seen for gentamicin (13.4% decrease). The only increase in resistance was seen for colistin (from 0.7% to 6.0% during COVID-19, p<.001) (Table 4).

**Table 4:** Resistance patterns for P. aeruginosa before and during COVID-19. * Statistically significant

Antibiotics	Before COVID-19		During COVID-19		p-value
	R (%)	S (%)	R (%)	S (%)	
Ampicillin	100	0	100	0	-
Amoxiclav	50.7	49.3	35.1	64.9	< .001*
Piperacillin-Tazobactam	28.6	71.4	11.0	89.0	< .001*
Cephalothin	54.4	45.6	40.1	59.9	< .001*
Cefuroxime	51.3	48.7	36.2	63.8	< .001*
Ceftazidime	46.6	53.4	31.8	68.2	< .001*
Cefoxitin	22.4	77.6	25.3	74.7	.103
Cefepime	45.2	54.8	28.2	71.8	< .001*
Cefotaxime	48.3	51.7	20.3	79.7	< .001*
Ceftriaxone	48.0	52.0	17.3	82.7	< .001*
Ciprofloxacin	31.8	68.2	27.2	72.8	.017*
Levofloxacin	24.6	75.4	15.4	84.6	< .001*
Gentamicin	23.2	76.8	14.1	85.9	< .001*
Amikacin	12.2	87.8	7.6	92.4	< .001*
Trimethoprim-Sulfamethoxazole	43.2	56.8	16.4	83.6	< .001*
Imipenem	22.1	77.9	12.6	87.4	< .001*
Meropenem	18.0	82.0	8.4	91.6	< .001*
Ertapenem	18.5	81.5	37.4	62.6	< .001*
Tigecycline	33.9	66.1	15.5	84.5	.003*
Nitrofurantoin	54.1	45.9	53.4	46.6	.801
Aztreonam	25.0	75.0	33.8	66.2	< .001*

**Figure 4:**
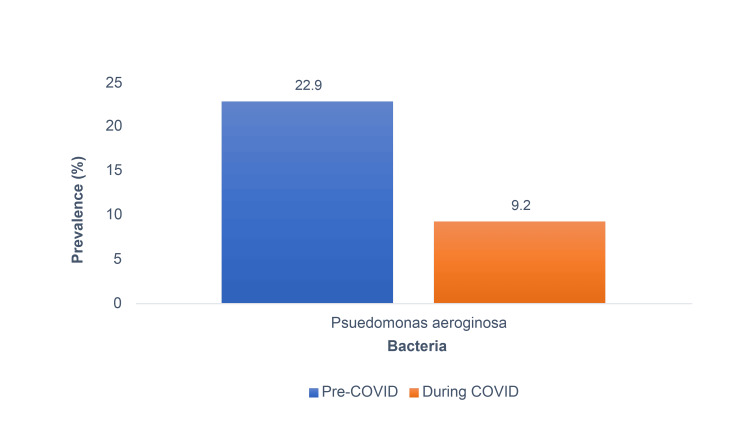
Prevalence of MDR among P. aeruginosa strains based on pre- COVID-19 and during COVID-19.

The overall prevalence of MDR among *P. aeruginosa *saw a decline from 22.9% pre-COVID-19 to 9.2% during COVID-19 (Figure 4). Results of the regression analysis model revealed that only one variable was associated with MDR with statistical significance. MDR was 66% less likely during COVID-19 as compared to prior to COVID-19 (p<.001) (Table 5).

**Table 5:** Results of regression analysis for predicting MDR among P. aeruginosa. ^a^ Logistic regression analysis. ^b^ Linear regression analysis. * Statistically significant.

Variable	OR (95% CI)	p-value
Male Gender^a^	0.83 (0.59 – 1.18)	0.309
Age^b^	0.01 (-0.01 – 0.001)	0.764
Inpatient^a^	0.53 (0.25 – 1.10)	0.088
During COVID-19^a^	0.34 (0.24 – 0.48)	< .001*

## Discussion

Discussing trends in AMR is crucial for understanding the factors that may influence its fluctuation over time. The COVID-19 pandemic significantly altered infection management and preventive measures, impacting AMR trends.

*E. coli*, a common pathogen in UTIs, has shown varied AMR trends. Prior to the pandemic, AMR rates were relatively high. A study from Ecuador observed a 42% reduction in resistance to third-generation cephalosporins during the pandemic [28]. Similarly, a multinational European study [29] found lower AMR rates during the COVID-19 pandemic compared to pre-pandemic levels. However, a recent U.S. study conducted in 2024 reported that AMR rates have risen again in the post-COVID era, indicating a resurgence in resistance levels [30]. Furthermore, a 2022 study comparing ICU and non-ICU infections documented persistently high levels of AMR [31].

*K. pneumoniae*, another frequent UTI pathogen, also demonstrated a notable drop in AMR trends from 2018 to 2022, though resistance to ampicillin remained steady. Pre-COVID data from Pakistan showed resistance to amoxiclav, imipenem, ciprofloxacin, and trimethoprim-sulfamethoxazole being 64%, 15%, 47.5%, and 51%, respectively [32]. Post-COVID studies from India in 2021 and 2022, including one on diabetic patients, reported resistance rates higher than those observed in more recent years [33].

*P. aeruginosa *showed an interesting trend; while most resistance rates dropped, colistin resistance increased. Post-COVID, resistance levels were generally lower by about 10% compared to other studies [34, 35]. A longitudinal study from the UAE between 2010 and 2021 showed only a minor decrease of 2.5% in AMR, despite the generally low resistance rates [36]. Additionally, during the pandemic, a drop in MDR *P. aeruginosa* was observed, similar to findings from a Polish study conducted between 2011 and 2018, which reported a 21.9% prevalence of MDR which was similar to our results of 22.9% [37]. However, a 2022 study in France reported a higher prevalence of 25% MDR in *P. aeruginosa* isolates from patients with UTI and asymptomatic bacteriuria [35].

The decrease in resistance observed for these pathogens during the pandemic might be attributed to several factors. Enhanced regulations on antibiotic prescriptions and improved patient compliance with treatment regimens likely played significant roles [38]. Additionally, social distancing measures reduced infection transmission rates, though AMR rates showed a slight increase in 2022 when these measures were eased. Increased public awareness and adherence to hand hygiene and sanitization practices might also have contributed to the observed trends [39].

Strengths and limitations

This is the first Saudi study to analyze AMR and MDR in urine samples across two distinct periods: before and during the COVID-19 pandemic. With a substantial sample size of 10,031 patient urine samples, the research provides robust evidence that is also in line with the World Health Organization’s Global Action Plan. This alignment emphasizes the importance of continual monitoring of AMR to effectively combat its spread, thus enhancing the study's relevance and contribution to a broader comprehension of AMR trends amid a global health crisis. The insights gained from examining AMR and MDR trends, especially in the context of a pandemic, lay a solid foundation for future research and the development of targeted antibiotic stewardship and public health strategies. However, the study is not without its limitations. Its retrospective design may introduce biases linked to data availability, completeness, and potential selection biases, which could affect the reliability of the findings. The use of exclusion criteria, while essential for the integrity of the study, could limit the applicability of the results to a wider population. The fact that the research was conducted in a single tertiary hospital further narrows its generalizability to other settings or geographic areas. Additionally, the specific temporal classification into pre-COVID-19 and during COVID-19 may not fully capture the nuanced impacts of the pandemic's varying phases and healthcare responses. Particularly, the study's focus on *P. aeruginosa* for MDR analysis, due to incomplete data on other bacteria, significantly restricts understanding of MDR across a more extensive range of pathogens. Potential confounders, such as changes in antibiotic prescribing practices and other external factors not considered in the study design, could also skew the resistance patterns and interpretations drawn from the results. These limitations highlight the need for careful consideration when extrapolating these findings to broader contexts.

## Conclusions

The findings of this study highlight significant shifts in antimicrobial resistance patterns among uropathogenic bacteria during the COVID-19 pandemic. The data showed a general trend of decreasing resistance to several antibiotics among *E. coli *and *K. pneumoniae *strains during COVID-19, although there were notable exceptions such as increased resistance to cefoxitin and imipenem. Likewise, *P. aeruginosa *strains exhibited decreased resistance to various antibiotics during COVID-19, except for colistin, which showed an increase in resistance patterns. These findings suggest that the dynamics of antimicrobial resistance have been influenced by the pandemic, possibly due to changes in antibiotic usage, healthcare practices, or patient populations. The observed decrease in MDR among* P. aeruginosa* strains during COVID-19 further emphasizes the complex interplay between infectious diseases, antimicrobial treatments, and public health emergencies. Considering healthcare concerns such as pandemics, further research is needed to identify the exact mechanisms and reasons behind these changes in resistance patterns to help devise targeted antimicrobial resistance management practices.
